# Myeloid deficiency of the intrinsic clock protein BMAL1 accelerates cognitive aging by disrupting microglial synaptic pruning

**DOI:** 10.1186/s12974-023-02727-8

**Published:** 2023-02-24

**Authors:** Chinyere Agbaegbu Iweka, Erica Seigneur, Amira Latif Hernandez, Sur Herrera Paredes, Mica Cabrera, Eran Blacher, Connie Tsai Pasternak, Frank M. Longo, Luis de Lecea, Katrin I. Andreasson

**Affiliations:** 1 Department of Neurology and Neurological Sciences, Stanford School of Medicine, Stanford, CA USA; 2 Department of Psychiatry and Behavioral Sciences, Stanford University, Stanford, CA USA; 3 Department of Biology, Stanford University, Stanford, CA USA; 4 Department of Biological Chemistry, The Alexander Silberman Institute of Life Sciences, The Hebrew University of Jerusalem, Edmond J. Safra Campus Givat-Ram, 9190401 Jerusalem, Israel; 5 Wu Tsai Neurosciences Institute, Stanford University, Stanford, CA USA; 6 Stanford Immunology Program, Stanford University, Stanford, CA USA; 7 Chan Zuckerberg Biohub, San Francisco, CA 94158 USA

**Keywords:** Circadian clock, BMAL1, Microglia, Synaptic plasticity, Sleep–wake cycle

## Abstract

Aging is associated with loss of circadian immune responses and circadian gene transcription in peripheral macrophages. Microglia, the resident macrophages of the brain, also show diurnal rhythmicity in regulating local immune responses and synaptic remodeling. To investigate the interaction between aging and microglial circadian rhythmicity, we examined mice deficient in the core clock transcription factor, BMAL1. Aging *Cd11b*^*cre*^*;Bmal*^*lox/lox*^ mice demonstrated accelerated cognitive decline in association with suppressed hippocampal long-term potentiation and increases in immature dendritic spines. C1q deposition at synapses and synaptic engulfment were significantly decreased in aging *Bmal1*-deficient microglia, suggesting that BMAL1 plays a role in regulating synaptic pruning in aging. In addition to accelerated age-associated hippocampal deficits, *Cd11b*^*cre*^*;Bmal*^*lox/lox*^ mice also showed deficits in the sleep–wake cycle with increased wakefulness across light and dark phases. These results highlight an essential role of microglial BMAL1 in maintenance of synapse homeostasis in the aging brain.

**Supplementary Information:**

The online version contains supplementary material available at 10.1186/s12974-023-02727-8.

## Background

The circadian clock is a time-keeping system that enables anticipation and adaptation to changes in the environment to maintain homeostasis in living organisms [1, 2]. Disruption of the circadian clock at the whole-body or organ level is associated with cardiovascular, metabolic, neoplastic and neurological disorders, and is a prominent feature in aging in many organ systems [3–7].

A central regulator of the cellular clock machinery is BMAL1 (brain and muscle Arnt (aryl hydrocarbon receptor nuclear translocator)-like protein) which dimerizes with CLOCK (circadian locomotor output cycles kaput) to regulate circadian gene expression in a cell-specific manner, where approximately 15% of all genes are rhythmically regulated within all cell types [8, 9]. BMAL1 is a member of the basic-helix–loop–helix (bHLH)-PER–ARNT–SIM (PAS) superfamily of transcription factors and was originally characterized by its high expression in the brain and muscle [10]. Global deletion of *Bmal1* accelerates age-related pathologies such as atherosclerosis, calcification of limb joints, and ocular abnormalities, and reduces lifespan [11]. In peripheral macrophages, *Bmal1* deletion disrupts the diurnal rhythmicity of immune cell trafficking, mitochondrial metabolism and inflammatory responses [12–14].

Microglia are brain-resident macrophages that maintain tissue homeostasis by clearing cellular debris and misfolded proteins and protecting against pathogens. Microglia also regulate synaptic circuitry by pruning non-functional synapses [15–17]. Several studies have demonstrated circadian rhythmicity in microglial expression of inflammatory molecules, synaptic pruning and changes in morphology that are altered with age [18–22]. However, the role of circadian regulation of microglial function in age-associated cognitive decline has not been explored. Here, we investigated the effects of circadian clock disruption in aging microglia by partial ablation of the core clock protein BMAL1 in myeloid-lineage cells. We find that microglia deficient in BMAL1 show disrupted synaptic pruning and a persistence of immature dendritic spines, a phenotype associated with accelerated age-related hippocampal memory decline and disrupted sleep–wake cycle.

## Results

### Microglial BMAL1 deficiency disrupts hippocampal-dependent memory in aged mice

**Fig. 1:**
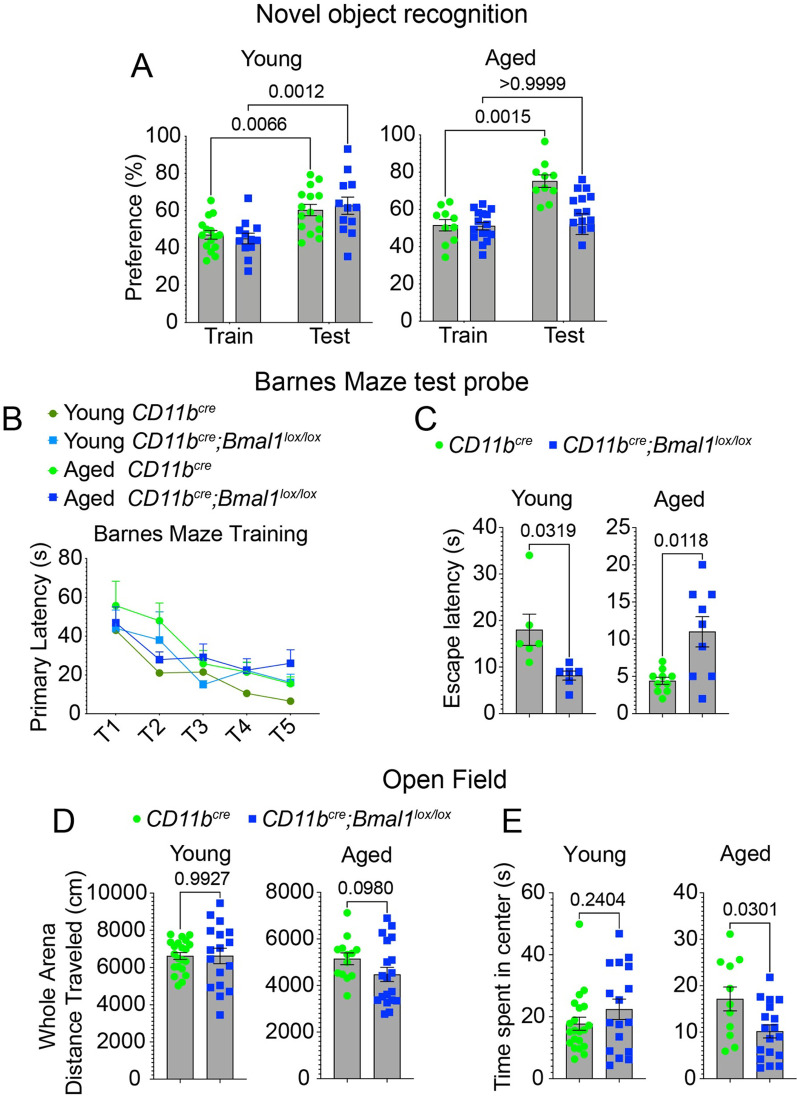
Microglia BMAL1 deficiency disrupts hippocampal-dependent behavior and increases anxiety in aged, but not young mice. **A** Preference in the novel object recognition task in young (3–6 months, *CD11b*^*cre*^, *n* = 15; *CD11b*^*cre*^*;Bmal1*^*lox/*lox^, *n* = 12) and aged (18–20 months, *CD11b*^*cre*^, *n* = 10; *CD11b*^*cre*^*;Bmal1*^*lox/lox*^, *n* = 16) mice. **B** Primary escape latency during the 5-day trial period for young and aged mice. **C** Escape latency in the testing phase of the Barnes maze task in young (3–6 months, *CD11b*^*cre*^, *n* = 6; *CD11b*^*cre*^*;Bmal1*^*lox/lox*^, *n* = 6) and aged (18–20 months, *CD11b*^*cre*^, *n* = 9; *CD11b*^*cre*^*;Bmal1*^*lox/lox*^, *n* = 10) mice. **D** Distance travelled in whole arena in the open field task in young (3–6 months, *CD11b*^*cre*^, *n* = 21; *CD11b*^*cre*^*;Bmal1*^*lox/lox*^, n = 17) and aged (18–20 months, *CD11b*^*cre*^*,* n = 12; *CD11b*^*cre*^*;Bmal1*^*lox/lox*^, *n* = 19) mice. **E** Time spent in the center area in the open field task in young (3–6 months, *CD11b*^*cre*^, n = 21; *CD11b*^*cre*^*;Bmal1*^*lox/lox*^, *n* = 17) and aged (18–20 months, *CD11b*^*cre*^*,* n = 12; *CD11b*^*cre*^*;Bmal1*^*lox/lox*^, *n* = 19) mice. Data are represented as the mean ± SEM. *P*-values were calculated using paired *t*-test, or two-tailed Student’s* t*-test.

The circadian clock declines with aging and is associated with deficits in learning and memory [6, 23, 24]. We have previously shown that macrophage circadian gene expression and immune responses decline in aging mice [25]. Aging microglia also exhibit a diminished capacity for normal function that is associated with impaired cognition [26–28]. To investigate the interaction between microglial circadian function and aging, we generated myeloid-specific *Bmal1* conditional knock out mice (*CD11b*^*cre*^*;Bmal1*^*lox/lox*^; cKO) and confirmed reduced BMAL1 protein expression in peritoneal macrophages (Additional file 1: Fig. S1A; *p* = *0.0178*). We then subjected young and aged (*CD11b*^*cre*^) WT and *Bmal1* cKO mice to a series of cognitive tasks. The novel object recognition task, a non-spatial recall test that measures discrimination between a familiar and novel object, revealed impaired memory in aged *Bmal1* cKO compared to littermate WT mice (Fig. 1A). Young *Bmal1* cKO mice performed similarly to WT in their examination of the novel object (Fig. 1A). Spatial learning and memory, which is encoded primarily by the hippocampus, was tested using the Barnes maze task. Primary escape latency over the 5-day training period decreased by a larger degree for young WT compared to aged WT and *Bmal1* cKO mice (Fig. 1 B). Aged *Bmal1* cKO showed a significant increase in escape latency compared to WT mice (Fig. 1C; *p* = *0.0118*), indicating a deficit in spatial memory. Young *Bmal1* cKO performed better on the Barnes maze task compared to WT mice (Fig. 1C; *p* = *0.0319*) consistent with a recent study showing that microglial loss of *Bmal1* improved cognition in young mice [29]. In the open field test, which assesses motor function, exploratory activity, and anxiety, there were no differences in motor function but there was increased anxiety in aged *Bmal1* cKO compared to WT mice (Fig. 1D, E; *p* = *0.0301*). There were no differences in motor activity, exploration or anxiety between young *Bmal1* cKO and WT mice (Fig. 1D, E). These results indicate that microglial BMAL1 deficiency accelerates age-associated deficits in spatial and non-spatial learning and memory.

### Microglial BMAL1 deficiency suppresses long-term potentiation in the CA1 hippocampal region of aged mice

**Fig. 2:**
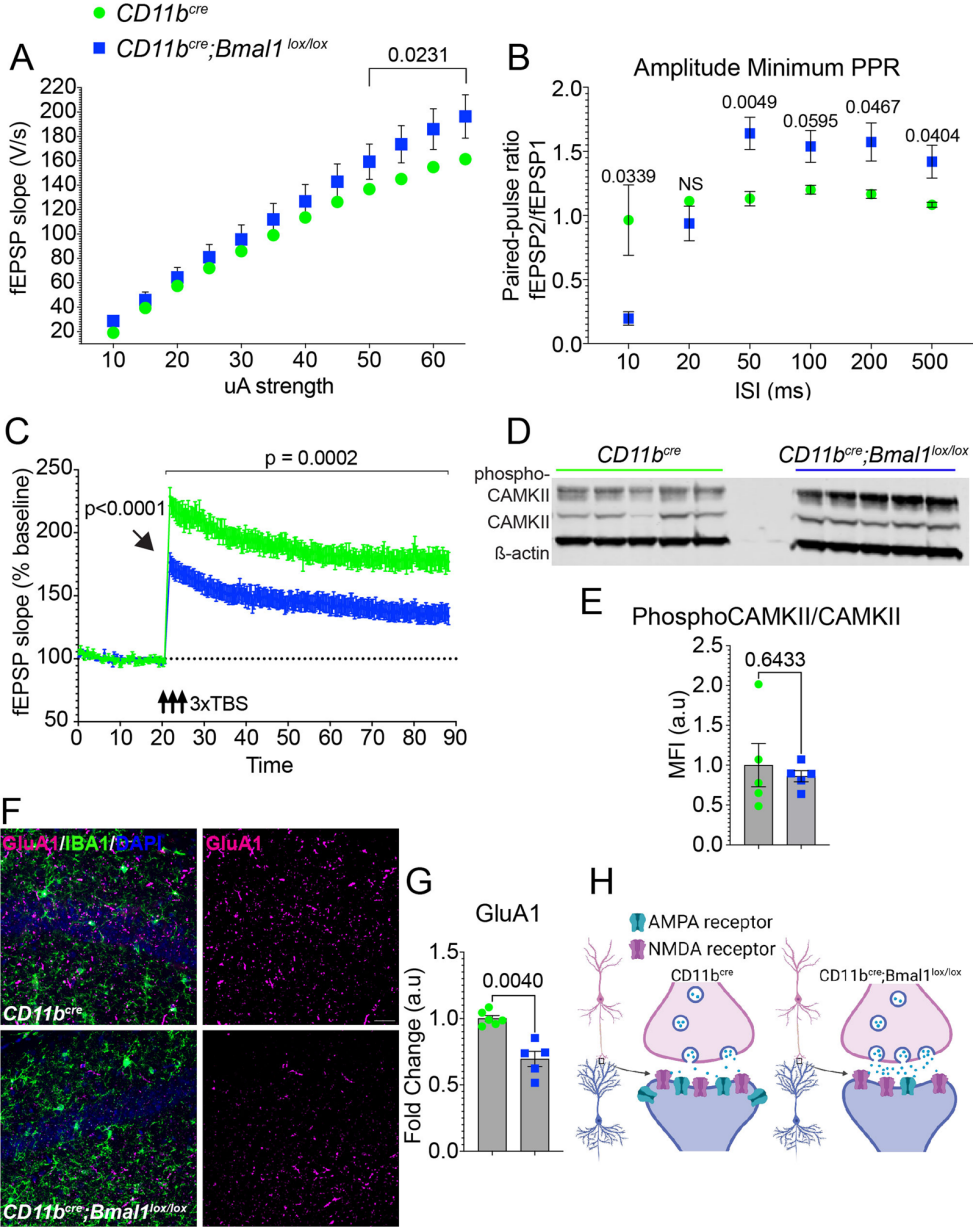
Deficits in long-term potentiation in the CA1 hippocampal region in aged *Bmal1* cKO mice. **A** Input/output (I/O) curves as a measure of basal synaptic transmission in the CA1 region of the hippocampus (10 slices, 5 mice per aged *CD11b*^*cre*^ and *CD11b*^*cre*^*;Bmal1*^*lox/lox*^ mice). **B** Paired-pulse ratio was recorded from CA1 pyramidal neurons with inter-stimulus intervals: 10, 20, 50, 100, 200, or 500 ms from aged *CD11b*^*cre*^ (18–20 months; *n* = 8) and *CD11b*^*cre*^*;Bmal1*^*lox/lox*^ (*n* = 10) mice. **C** Long-term potentiation (LTP) in the CA1 hippocampal region over a 90-min recording interval (*n* = 10 slices, 5 mice per aged *CD11b*^*cre*^ and *CD11b*^*cre*^*;Bmal1*^*lox/lox*^ mice). Arrow shows that induction of LTP is significantly reduced in *CD11b*^*cre*^ (228.669 ± 7.285) and *CD11b*^*cre*^*;Bmal1*^*lox/lox*^ (176.359 ± 7.593; *p* < *0.0001*). **D** Representative immunoblot of phospho-CAMKII and CAMKII in aged *CD11b*^*cre*^ and *CD11b*^*cre*^*;Bmal1*^*lox/lox*^ mice (18–20 months; n = 5/group). **E** Quantification of phospho-CAMKII and CAMKII immunoblot in **D** normalized to ß-actin. **F** Representative confocal images of GluA1 (magenta) expression in the hippocampal CA1 region of aged *CD11b*^*cre*^ (top, *n* = 6) and *CD11b*^*cre*^*;Bmal1*^*lox/lox*^ (bottom, *n* = 5) mice. Scale bar, 50 µm. **G** Quantification of mean fluorescence intensity of GluA1 in the hippocampal CA1 region of aged *CD11b*^*cre*^ (n = 6) and *CD11b*^*cre*^*;Bmal1*^*lox/lox*^ (*n* = 5) mice. **H** Diagram highlighting differences in synaptic release and numbers of receptors between genotypes in aged CA1 hippocampus. Data are represented as the mean ± SEM. *P*-values were calculated using two-way RM ANOVA or two-way ANOVA: effects of time and genotype and Sidak’s multiple comparisons test with Geisser–Greenhouse correction. Theta burst stimulation (TBS)

Next, we asked if neural correlates of learning and memory in aged mice were altered in *Bmal1* cKO mice. First, we assessed basal synaptic transmission. The input/output (I/O) curve reflecting the efficacy of pre- and post-synaptic neurotransmitter release was significantly increased at higher stimulation strengths (50 to 65 µA) in aged *Bmal1* cKO mice, consistent with increased basal synaptic transmission (Fig. 2A; *p* = *0.0231*). Next, we evaluated short-term synaptic plasticity by measuring paired-pulse ratio (PPR) in the CA1 region of the hippocampus. PPR measures the probability of activity-dependent presynaptic vesicular release following an action potential [30]. The ratio of the amplitude of the second response to that of the first to stimulation is inversely related to the release probability. PPR was significantly reduced at 10 ms (Fig. 2B; *p* = *0.0339*) and increased at 50 ms (*p* = *0.0049*), 100 ms (*p* = *0.0595*), 200 ms (*p* = *0.0467*), and 500 ms (*p* = *0.0404*) in aged *Bmal1* cKO compared to WT mice (Fig. 2B), consistent with alterations in presynaptic short-term plasticity.

We next examined long-lasting, activity-dependent changes in synaptic efficacy from the CA3 to CA1 Schaffer collateral pathway in the hippocampus to assess postsynaptic plasticity. We found significant impairment in long-term potentiation (LTP) in hippocampal slices from aged *Bmal1* cKO compared to WT mice across 70 min of recording (Fig. 2C; main effect of genotype for 70 min post-induction: F_1,18_ = 20.77, *p* = *0.0002*). This impairment was apparent immediately after LTP induction (Fig. 2C; *p* < *0.0001*). Thereafter, LTP in aged *Bmal1* cKO mice slowly decayed to ~ 35% potentiation, whereas WT mice stabilized LTP at ~ 80% with respect to baseline.

Calcium/calmodulin-dependent protein kinase II (CaMKII) is critical for the induction of LTP, and inhibition of CAMKII prevents LTP [31–34]. To investigate the candidate mechanisms underlying the reduced LTP in aged *Bmal1* cKO mice, we tested whether microglial *Bmal1* deficiency might alter hippocampal levels of CAMKII or phosphorylated CAMKII. Quantitative immunoblotting showed increased total CAMKII expression and CAMKII phosphorylation, however, there was no difference in the ratio of phosphorylated CAMKII/total CAMKII between aged *Bmal1* cKO and WT mice (Fig. 2D, E; *p* = *0.6433*). The AMPA receptor subunit, GluA1, mediates LTP maintenance by enhancing AMPA receptor conductance and anchoring at the synapse [35]. To reconcile the increase in I/O ratio with decreased LTP induction and amplitude, we assessed levels of GluA1 expression. Aged *Bmal1* cKO mice exhibited decreased GluA1 signal in the CA1 hippocampal region compared to WT mice (Fig. 2F–H; *p* = *0.0040*). These data suggest that microglial BMAL1 deficiency alters hippocampal plasticity in aged mice by a mechanism involving reduced insertion of AMPA receptors in dendritic spines in aged mice.

### BMAL1-deficient microglia increase synaptic density in the CA1 hippocampal region of aged mice

**Fig. 3:**
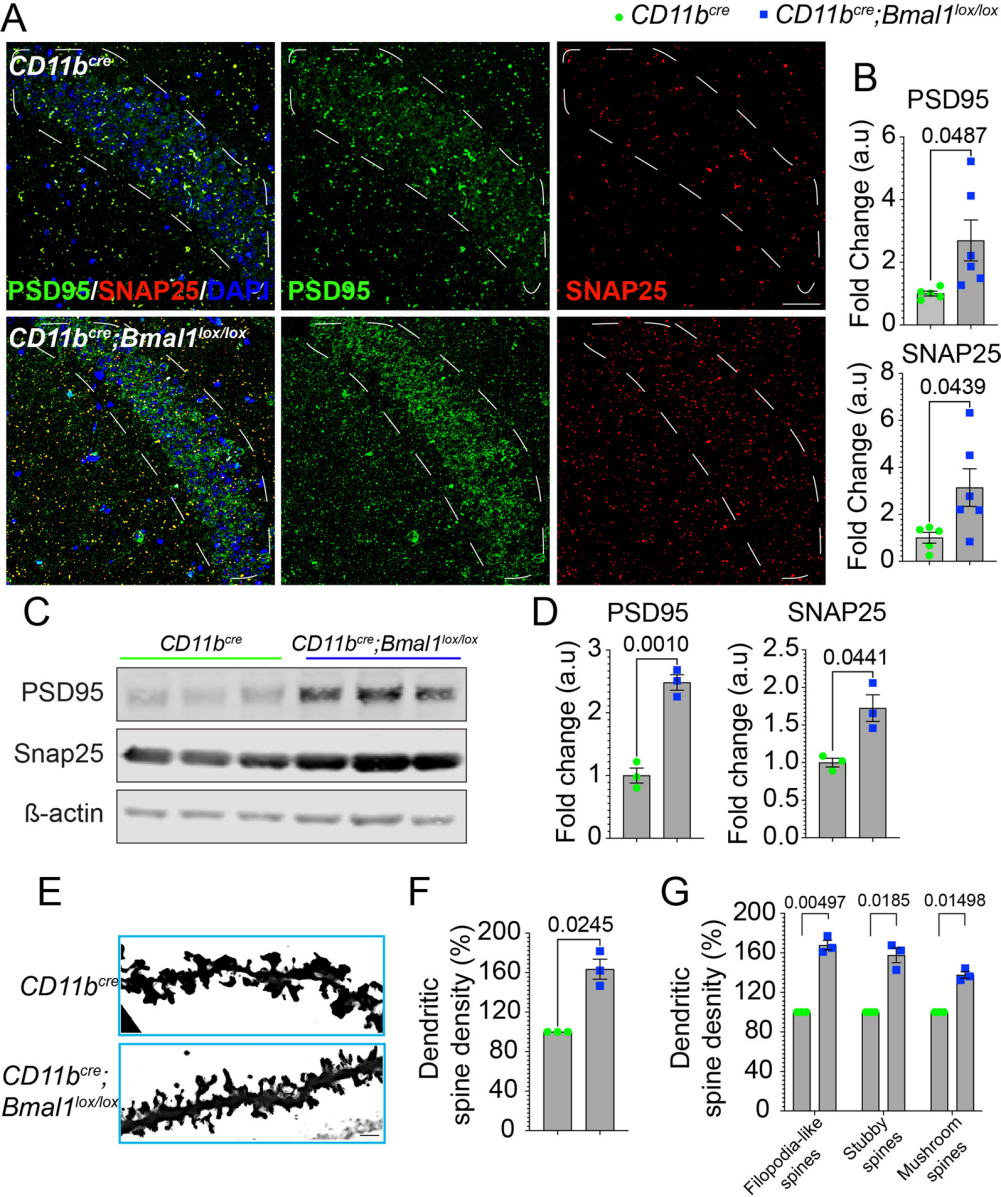
Increased numbers of immature spines in aged *Bmal* cKO CA1 hippocampus. **A** Representative images of PSD95 (green) and SNAP25 (red) expression in aged *CD11b*^*cre*^ and *CD11b*^*cre*^*;Bmal1*^*lox/lox*^ mice (18–20 months). Scale bar, 50 µm. White dotted lines in **A** indicate region of interest quantified in **B**. **B** Mean fluorescence intensity of PSD95 and SNAP25 in aged *CD11b*^*cre*^ (*n* = 5) and *CD11b*^*cre*^*;Bmal1*^*lox/lox*^ mice (*n* = 6). **C** Representative immunoblot of PSD95 and SNAP25 in aged *CD11b*^*cre*^ and *CD11b*^*cre*^*;Bmal1*^*lox/lox*^ mice (18–20 months; *n* = 3/group). **D** Quantification of PSD95 and SNAP25 normalized to ß-actin. **E** Representative images of Golgi stain of apical dendritic spines in the CA1 region of the hippocampus aged *CD11b*^*cre*^ and *CD11b*^*cre*^*;Bmal1*^*lox/lox*^ mice (18–20 months; n = 3/group). Scale bar, 5 µm. **F** Dendritic spine density (10–12 neurons/CA1 region) in aged *CD11b*^*cre*^ and *CD11b*^*cre*^*;Bmal1*^*lox/lox*^ (*n* = 3/group) mice. **G** Dendritic spine density of filopodia-like, stubby and mushroom spines in aged *CD11b*^*cre*^ and *CD11b*^*cre*^*;Bmal1*^*lox/lox*^ (*n* = 3/group) mice. Data are represented as the mean ± SEM. *P*-values were calculated using two-tailed Student’s* t*-test

Microglia actively maintain neural circuitry and plasticity through selective pruning of synapses [36–38]. Given the cognitive deficits and altered synaptic plasticity observed in aged *Bmal1* cKO mice, we measured expression levels of presynaptic and postsynaptic proteins, SNAP25 and PSD95, respectively, in the CA1 hippocampal region. Aged *Bmal1* cKO mice showed higher immunoreactivity for both PSD95 *(p* = *0.0487)* and SNAP25 *(p* = *0.0439)* compared to aged WT mice (Fig. 3A, B). No significant differences were observed between young *Bmal1* cKO and WT mice (Additional file 1: Fig. S1B, C). These results were confirmed using quantitative immunoblotting for PSD95 *(p* = *0.0010)* and SNAP25 *(p* = *0.0441)* (Fig. 3C, D). Assessment of synaptic morphology and density by Golgi staining confirmed increased dendritic spine density in aged *Bmal1* cKO compared to WT mice (Fig. 3E, F; *p* = *0.0245*). Aged *Bmal1* cKO mice had a higher density of filopodia-like *(p* = *0.00497)*, stubby *(p* = *0.0185)* and mushroom spines *(p* = *0.01498)* compared to WT mice (Fig. 3G), indicating a persistence of immature synapses in aged *Bmal1* cKO mice. These data suggest a significant impairment in pruning of synapses resulting from microglial BMAL1 deficiency in aged mice.

### Microglial BMAL1 deficiency decreases C1q expression and synaptic pruning in aged CA1 hippocampus

**Fig. 4:**
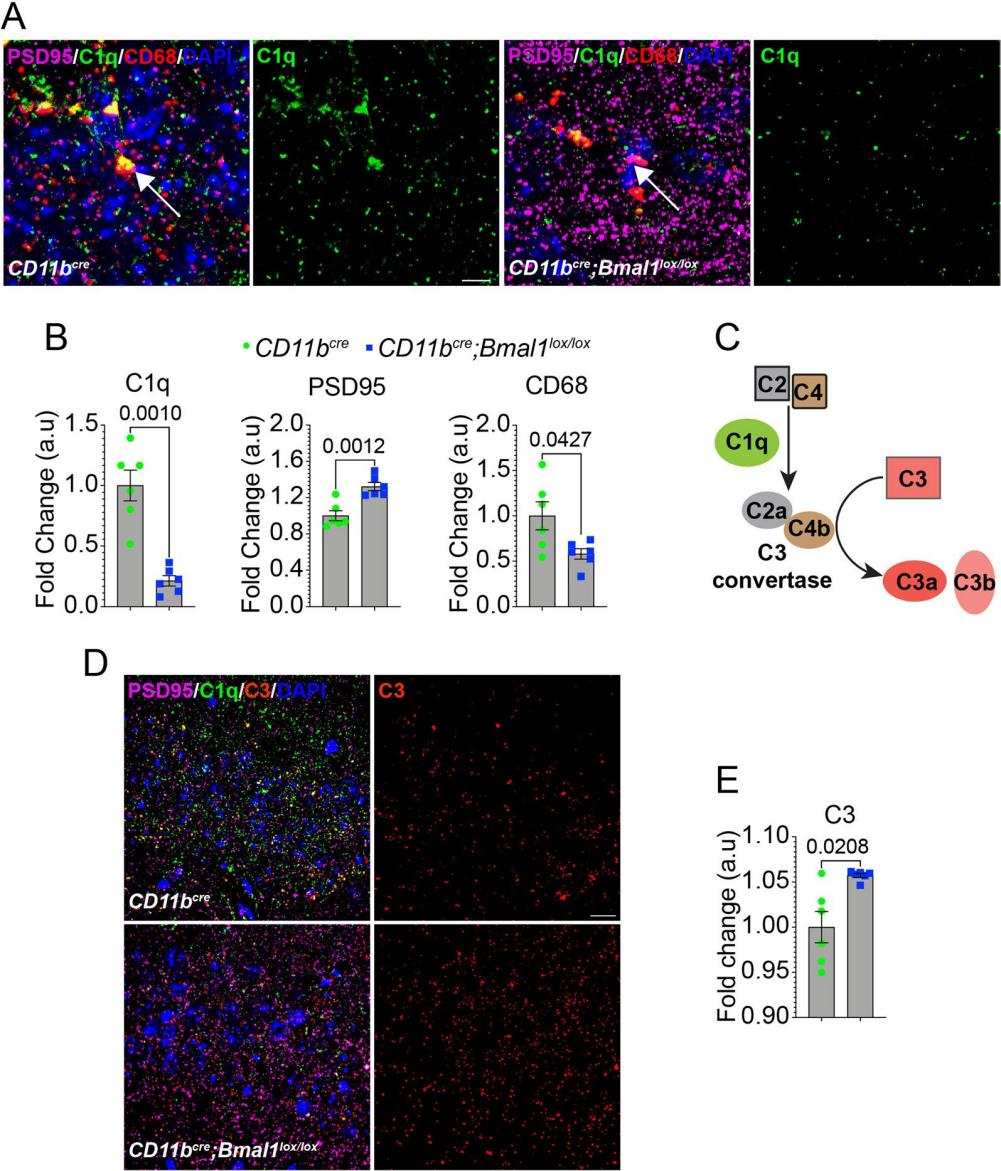
Microglial BMAL1 deficiency decreases C1q and increases C3 in aged CA1 hippocampus. **A** Representative images of C1q (green), PSD95 (magenta) and CD68 (red) expression in the hippocampal CA1 region of aged *CD11b*^*cre*^ and *CD11b*^*cre*^*;Bmal1*^*lox/lox*^ mice (18–20 months). Scale bar, 10 µm. White arrow shows colocalization of C1q, PSD95 and CD68 present in aged *CD11b*^*cre*^ that is absent in *CD11b*^*cre*^*;Bmal1*^*lox/lox*^ mice. **B** Mean fluorescence intensity (MFI) of C1q, PSD95 and CD68 (n = 6/group). **C** Schematic illustrating C1q activation of C3. **D** Representative confocal images of C1q (green), PSD95 (magenta) and C3 (red) expression in the CA1 hippocampal area in aged *CD11b*^*cre*^ and *CD11b*^*cre*^*;Bmal1*^*lox/lox*^ mice. Scale bar, 20 µm. **E** MFI of C3 in aged *CD11b*^*cre*^ and *CD11b*^*cre*^*;Bmal1*^*lox/lox*^ mice (n = 6/group). Data are represented as the mean ± SEM. *P*-values were calculated using two-tailed Student’s* t*-test

To investigate mechanisms that may underlie defective synaptic pruning in aged *Bmal1* cKO mice, we measured expression of C1q, an opsonin that is deposited on synaptic terminals and promotes synapse removal through interaction with the microglial C3 receptor, CR3 [37, 39, 40]. C1q is primarily expressed by microglia and increases with age, although neuronal expression has also been reported [39, 41, 42]. In the CA1 hippocampal region of aged *Bmal1* cKO mice, C1q colocalized with PSD95, but its abundance was significantly reduced (Fig. 4A, B; *p* = *0.0010*) in contrast to PSD95 levels (Fig. 4A, B; *p* = *0.0012)* which were significantly elevated (Fig. 3A, B). CD68, a microglial lysosomal protein, was observed in close proximity to PSD95/C1q and was also decreased in aged *Bmal1* cKO (Fig. 4A, B; *p* = *0.0427*), suggesting reduced microglial production of both C1q and CD68. There were no differences in C1q, PSD95 or CD68 levels between young *Bmal1* cKO and WT mice (Additional file 1: Fig. S2A, B).

C1q promotes the formation of C3 convertase through activation of complement proteins, C4 and C2. C3 convertase cleaves C3 to produce C3a, a pro-inflammatory mediator, and C3b, an opsonin that initiates phagocytosis via the CR3 receptor on microglia [43]. We observed a reciprocal increase in C3 immunoreactivity in the CA1 hippocampal region of aged *Bmal1* cKO mice compared to WT (Fig. 4C–E; *p* = *0.0208*), suggesting that C3 may accumulate in response to reduced levels of C3 convertase.

**Fig. 5:**
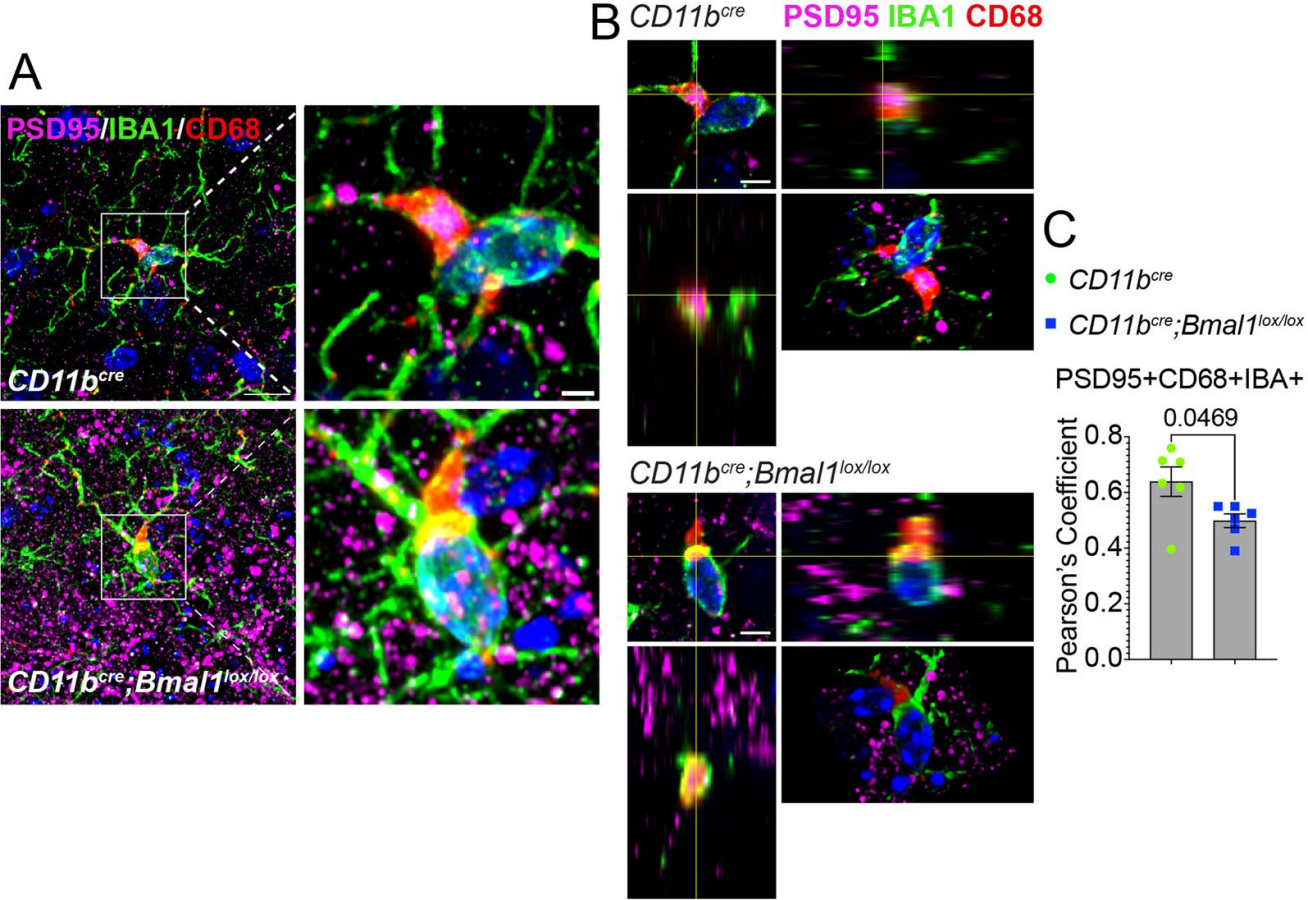
Microglial BMAL1 deficiency decreases synaptic engulfment in aged mice. **A** Representative images of IBA1 + /CD68 + cell engulfment of PSD95 + particles in microglia in aged *CD11b*^*cre*^* and CD11b*^*cre*^*;Bmal1*^*lox/lox*^ mice. Scale bar, 2 µm. **B** Orthogonal views and rotated 3D projection of PSD95 + particles in IBA + /CD68 + microglia from aged *CD11b*^*cre*^* and CD11b*^*cre*^*;Bmal1*^*lox/lox*^ mice*.* **C** Pearson’s coefficient of colocalization of PSD95 and CD68 in IBA1 + cells (n = 6/group) mice. Data are represented as the mean ± SEM. *P*-values were calculated using two-tailed Student’s* t*-test

Next, we assessed microglial engulfment of synapses by measuring colocalization of PSD95 and CD68 within IBA1 + microglia. Superresolution microscopy was used to quantify PSD95 within CD68 + structures in IBA + microglia in order to identify engulfed synapses. Aged *Bmal1* cKO microglia demonstrated a significant decrease in internalized PSD95 compared to WT (Fig. 5A–C; *p* = *0.0469*; Additional file 1: Video S1), consistent with decreased synapse engulfment. Taken together, these data indicate that microglial loss of BMAL1 decreases C1q deposition on synapses and synaptic engulfment.

### Microglia deficient in BMAL1 exhibit microgliosis and reduced expression of lysosomal proteins in the CA1 hippocampal region

**Fig. 6:**
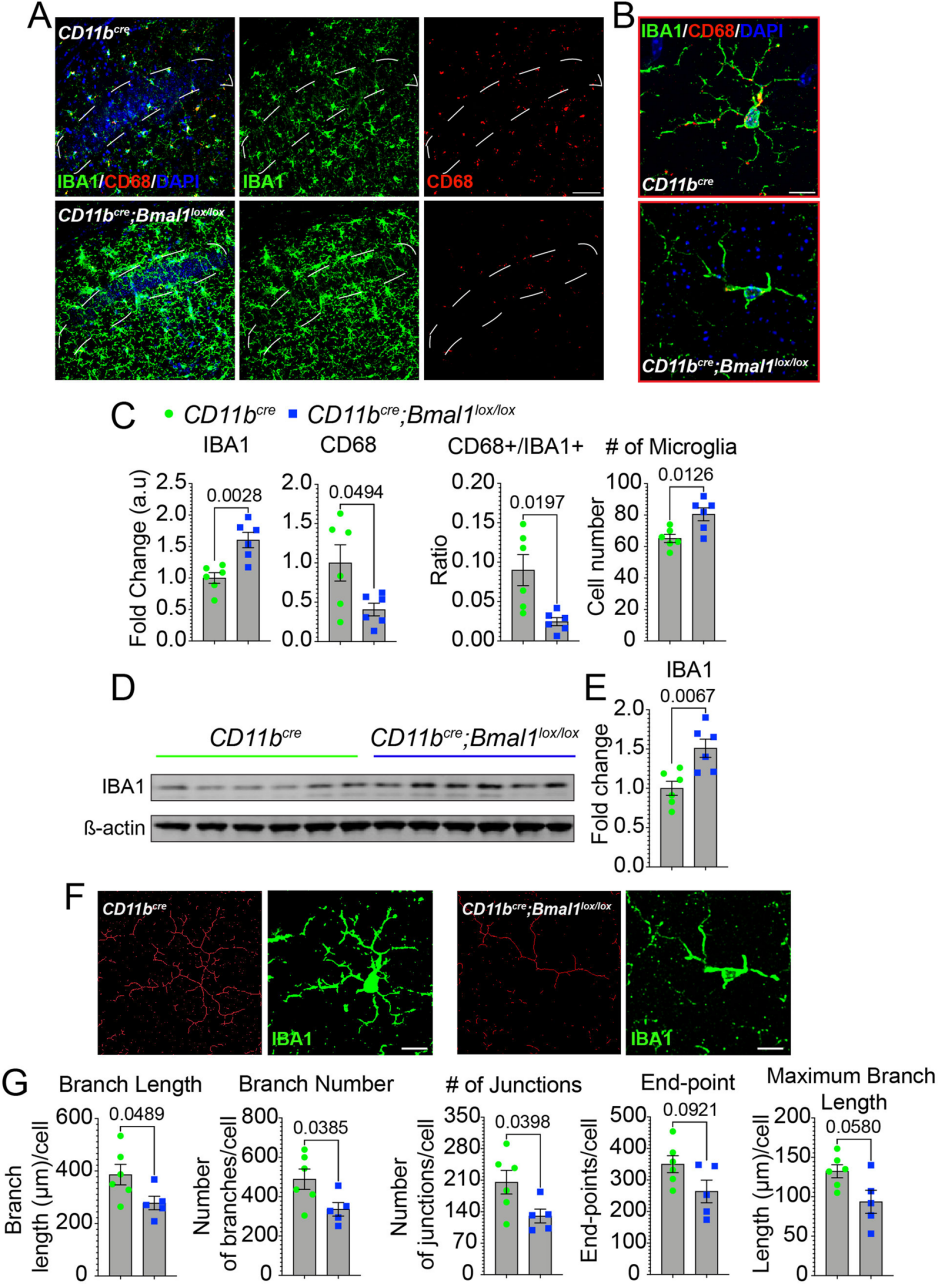
Myeloid *Bmal1* deletion increases microglial activation but decreases lysosomal function in aged mice.** A** Representative confocal images of IBA1 (green) and CD68 (red) expression in the hippocampal CA1 region of aged *CD11b*^*cre*^ and *CD11b*^*cre*^*;Bmal1*^*lox/lox*^ mice (18–20 months, n = 6/group). Scale bar, 50 µm. White dotted lines in **A** indicate region of interest quantified in **C**. **B** Higher magnification of microglia in CA1 hippocampal region from aged *CD11b*^*cre*^ and *CD11b*^*cre*^*;Bmal1*^*lox/lox*^ mice.Scale bar, 5 µm. **C** Mean fluorescence intensity of IBA1, CD68, proportion of IBA1-positive ( +) cells that are CD68 + , and the number of DAPI + IBA1 + microglia (n = 6/group). **D** Representative immunoblot of IBA1 in aged *CD11b*^*cre*^ and *CD11b*^*cre*^*;Bmal1*^*lox/lox*^ mice (18–20 months; *n* = 6/group). **E** Quantification of IBA1 immunoblot normalized to ß-actin. **F** Representative images of skeletonized microglia overlaid on original image from aged *CD11b*^*cre*^ and *CD11b*^*cre*^*;Bmal1*^*lox/lox*^ mice. **G** Quantification of microglial complexity. Scale bar, 5 µm. Every measurement that contained ≤ 2 endpoints with a maximum branch length of less than the cutoff value of 0.5 µm was removed from the analysis. Data are represented as the mean ± SEM. *P*-values were calculated using two-tailed Student’s* t*-test

To further characterize *Bmal1*-deficient microglia, we measured IBA1 and CD68 expression in the CA1 hippocampal region. A significant increase was observed in IBA1 immunoreactivity *(p* = *0.0028)* and number of microglia *(p* = *0.0126)* in aged *Bmal1* cKO compared to WT mice (Fig. 6A–C). Quantitative immunoblot analysis confirmed the increase in IBA1 expression in aged *Bmal1* cKO mice (Fig. 6D, E; *p* = *0.0067*). Consistent with our previous observation shown in Fig. 4B, CD68 was significantly decreased in aged *Bmal1* cKO mice (Fig. 6A–C; *p* = *0.0494*). The proportion of IBA1 + cells immunoreactive for CD68 was also significantly reduced, indicating an overall decrease in CD68 expression (Fig. 6A–C; *p* = *0.0197*). Morphological analysis of microglial complexity revealed decreases in branch length *(p* = *0.0489)*, branch number *(p* = *0.0385)* and number of junctions *(p* = *0.0398*) in aged *Bmal1* cKO compared to WT mice (Fig. 6F–G). No differences between young WT and *Bmal1* cKO mice in either IBA1 or CD68 expression, proportion of IBA1 + cells immunoreactive for CD68, number of microglia, or morphological parameters were observed (Additional file 1: Fig. S3A–E).

CD68 is a member of the lysosomal/endosomal-associated transmembrane glycoprotein (LAMP) family whose expression is upregulated with inflammatory stimuli such as bacterial lipopolysaccharide (LPS). Microglial CD68 expression and lysosomal dysfunction increases with aging and is associated with neurodegeneration [44–47]. We investigated additional lysosomal proteins including LAMP1, whose expression was similarly reduced in the CA1 hippocampal region of aged *Bmal1* cKO mice (Additional file 1: Fig. S4A, B; *p* = *0.0335*), as was p62 (Additional file 1: Fig. S4C, D; *p* = *0.0483*) which is required for the formation of the omegasome, a precursor structure of the autophagolysosome [48, 49]. These data suggest that although microglia in aged *Bmal1* cKO mice are proliferating and are morphologically activated, their reduced levels of CD68, LAMP1 and p62 suggest a deficit in lysosomal function.

### Deficiency of BMAL1 in microglia alters the omegasome and viral response genes

**Fig. 7:**
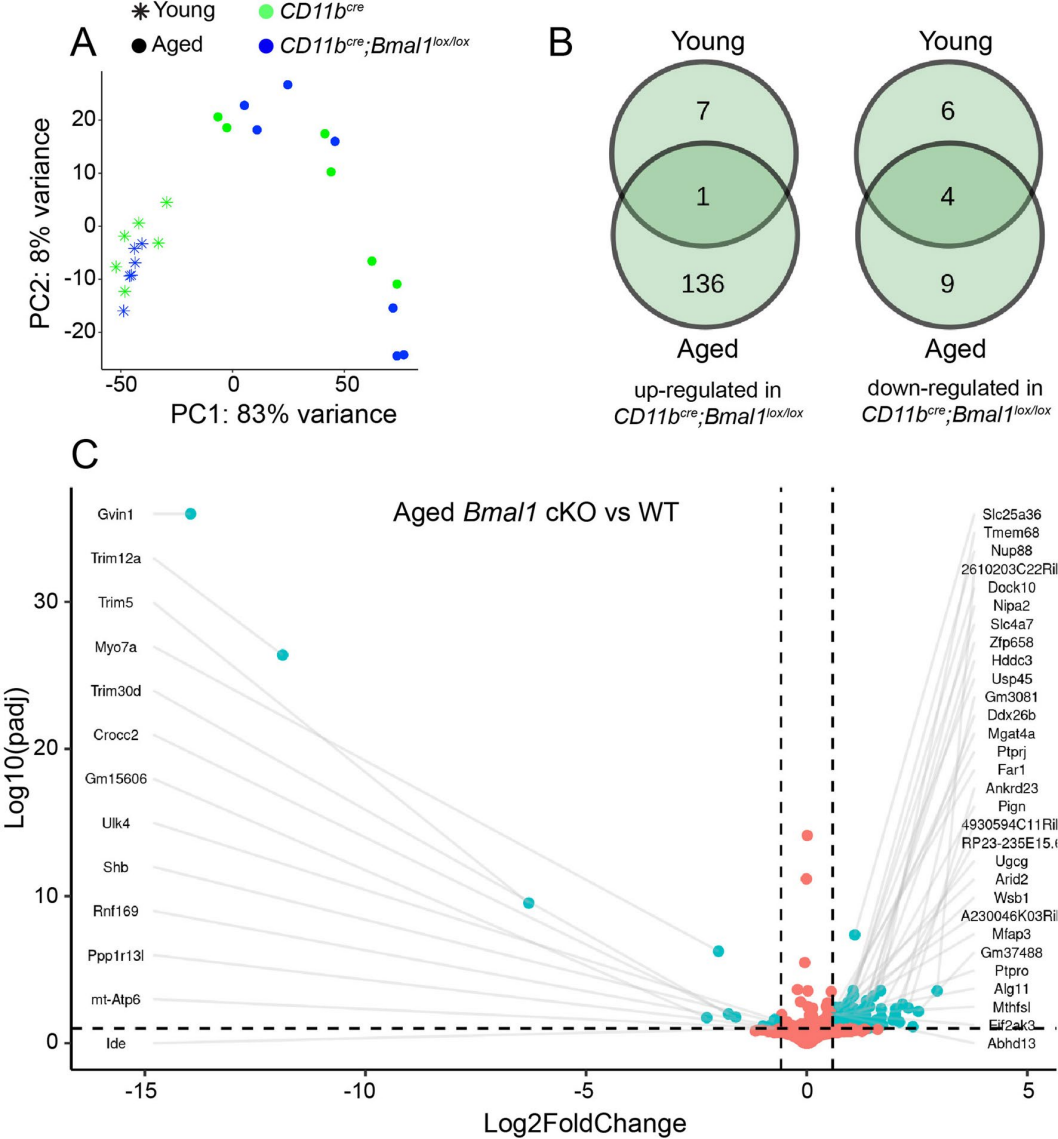
Microglial gene expression changes in young and aged WT and *Bmal1* cKO mice. **A** Principal component analysis (PCA) shows distinct clustering of young *CD11b*^*cre*^ and *CD11b*^*cre*^*;Bmal1*^*lox/lox*^ (*n* = 6/group) mice versus aged *CD11b*^*cre*^ (*n* = 6) and *CD11b*^*cre*^*;Bmal1*^*lox/lox*^ (*n* = 7) mice. **B** Venn diagram of number of differentially expressed genes in young and aged *CD11b*^*cre*^*;Bmal1*^*lox/lox*^ mice. **C** Volcano plot of -log10 p-adjusted value versus −log2 fold change of normalized counts between aged *CD11b*^*cre*^ and *CD11b*^*cre*^*;Bmal1*^*lox/lox*^ mice. Each dot represents a single transcript. Cyan dots denote significantly differentially expressed genes. Orange dots denote genes that are unchanged between the genotypes (adjusted *p*-value < 0.05)

To identify biological pathways affected by microglial loss of BMAL1, we performed RNA sequencing on CD11b + microglia isolated from young and aged, WT and *Bmal1* cKO mice. Principal component analysis (PCA) showed a large difference in transcriptional profiles driven by age (PC1) with variability among the aged mice (PC2) (Fig. 7A). No distinction between genotypes in either aged group was observed at the full transcriptome level. At the gene level, differential expression analysis (DEA) revealed 150 differentially expressed genes (DEGs) between *Bmal1* cKO and WT aged mice. 4/19 (21%) downregulated and 1/144 (< 1%) upregulated genes overlapped between young and aged *Bmal1* cKO mice (Fig. 7B). Of those, 13/150 (9%) were downregulated and 137/150 (91%) were upregulated (Fig. 7C). 18 DEGs were identified between young *Bmal1* cKO and WT mice, with 10/18 (56%) upregulated and 8/18 (44%) downregulated genes in *Bmal1* cKO (Additional file 1: Fig. S5A). Gene ontology (GO) analysis showed significant enrichment only in downregulated genes for biological functions that include innate immunity, autophagy and viral response (Additional file 2: Table S1). Interestingly, GO enrichment for cellular component was significant for the omegasome pathway, in line with our observations of decreased p62 protein levels in aged *Bmal1* cKO microglia.

### Microglial deficiency of BMAL1 disrupts the sleep–wake cycle

**Fig. 8:**
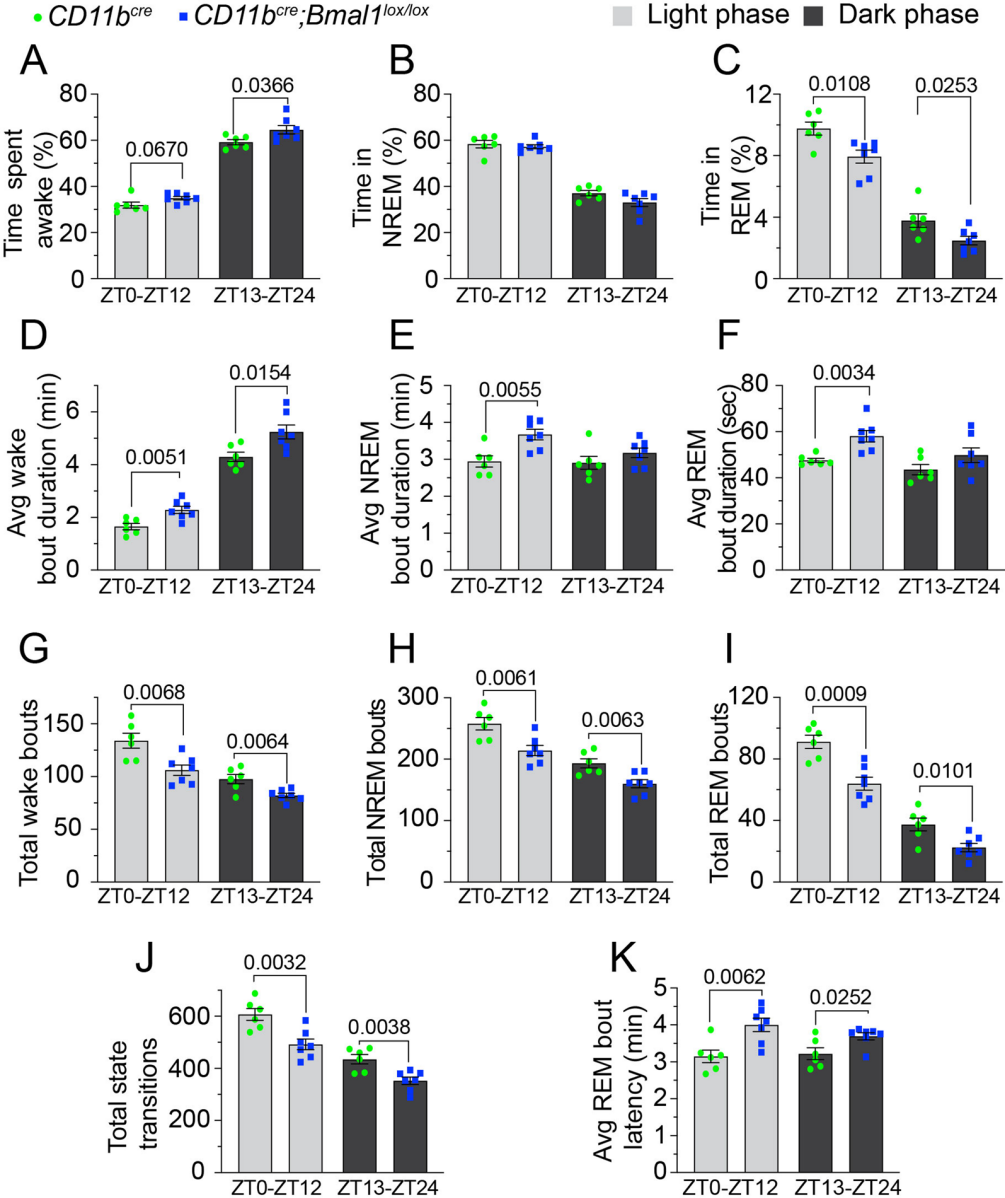
Sleep–wake behavior is disrupted in aged *Bmal1* cKO mice. Baseline sleep–wake behavior was assessed over continuous 24-h recordings of aged *CD11b*^*cre*^ (18–20 months; *n* = 6) and *CD11b*^*cre*^*;Bmal1*^*lox/lox*^ (*n* = 7) mice under normal light–dark (12-h light:12-h dark) conditions. **A**–**C** Percentage of time spent in wake (**A**), NREM sleep (**B**), and REM sleep (**C**) across the entire 12-h light/dark period. **D**–**F** The average duration of individual wake (**D**), NREM (**E**), and REM (**F**) bouts across the entire 12-h light/dark phase. **G**–**J** The total number of wake (**G**), NREM (**H**), and REM (**I**) bouts across the 12-h light/dark phases. **J** Transitions between sleep–wake states across the 12-h light/dark phases. **K** The average REM bout latency during the 12-h light/dark periods. Data are represented as the mean ± SEM. *P*-values were calculated using two-tailed Student’s* t*-test or two-way ANOVA. *ZT* zeitgeber time

Sleep is a major regulator of microglial function, and neuron–microglia interactions change during wake, NREM and REM states, reflecting changes in synapse remodeling [50]. Indeed, the sleep–wake cycle drives changes in synaptic ultrastructure and the daily dynamics of synaptic protein phosphorylation relevant to spine dynamics [51, 52]. The circadian clock plays a substantial role in sleep architecture, and global loss of BMAL1 disrupts sleep–wake patterns [53, 54]. Therefore, we carried out continuous 24-h electroencephalogram/electromyogram (EEG/EMG) recordings to determine baseline sleep–wake patterns in aged *Bmal1* and WT mice over 3 days. A trend towards increased wakefulness was observed in the *Bmal1* cKO mice (Fig. 8A–C). During the non-active light phase/subjective night, aged *Bmal1* cKO mice spent less time in REM sleep (Fig. 8C; *p* = 0.0108) compared to WT mice. Similarly, during the active dark phase/subjective day, aged *Bmal1* cKO mice spent more time awake (Fig. 8A; *p* = 0.0366) and less time in REM sleep (Fig. 8C; *p* = 0.0253).

During both the light and dark phases, aged *Bmal1* cKO mice exhibited an overall trend towards prolonged sleep–wake cycling. For example, during the non-active light phase/subjective night, the average duration of individual wake (Fig. 8D; *p* = 0.0051), NREM (Fig. 8E; *p* = 0.0055), and REM (Fig. 8F; *p* = 0.0034) bouts was increased, while the total number of wake (Fig. 8G; *p* = 0.0068), NREM (Fig. 8H; *p* = 0.0061), and REM (Fig. 8I; *p* = 0.0009) bouts, and transitions between all sleep–wake states (Fig. 8J; *p* = 0.0032) were decreased in the aged *Bmal1* cKO mice compared to WT. Similarly, during the active dark phase/subjective day, the average wake bout duration was increased (Fig. 8D; *p* = 0.0154) and the total number of wake bouts (Fig. 8G; *p* = 0.0064), NREM bouts (Fig. 8H; *p* = 0.0063), REM bouts (Fig. 8I; *p* = 0.0101), and state transitions (Fig. 8J; *p* = 0.0038) was decreased in the aged *Bmal1* cKO mice. Additionally, the average REM bout latency (time from NREM onset to REM onset) was increased in the aged *Bmal1* cKO mice during both the light (Fig. 8K; *p* = 0.0062) and dark (Fig. 8K; *p* = 0.0252) phases compared to WT.

Next, we performed an EEG spectral power analysis by averaging the relative values of delta 1 (0.5–2 Hz), delta 2 (2.5–4.5 Hz), theta (5–9 Hz), alpha (6–10 Hz) and beta (15.5–20 Hz) activity during wake, NREM, and REM sleep during the non-active light phase/subjective night. Aged *Bmal1* cKO mice exhibited a relative shift in spectral power characteristics during the wake period, with delta 1 power significantly decreased *(p* = *0.0084)*, and theta power increased *(p* = *0.0144)*, compared to aged WT mice (Additional file 1: Fig. S6A). No differences were observed during NREM and REM periods (Additional file 1: Fig. S6B, C*).* In humans, increased delta power and decreased theta power during wake has been correlated with subjective feelings of sleepiness [55, 56], and in mice the wake-promoting drug modafinil decreases delta power and increases theta power during wakefulness [57, 58]. Taken together, the observed decreased delta and increased theta power, which are signatures of lower sleep pressure, suggest increased wakefulness in the aged *Bmal1* cKO mice. Interestingly, the delta 2 EEG band, which is also associated with homeostatic sleep pressure [59] was unaffected in the *Bmal1* cKO mice (Additional file 1: Fig. S6).

These results indicate increased wakefulness during both light and dark periods in aged *Bmal1* cKO mice and suggest a role of circadian-tuned microglia in regulating overall neuronal excitability during the sleep–wake cycle.

### Microglial deficiency of BMAL1 cKO alters response to sleep deprivation

**Fig. 9:**
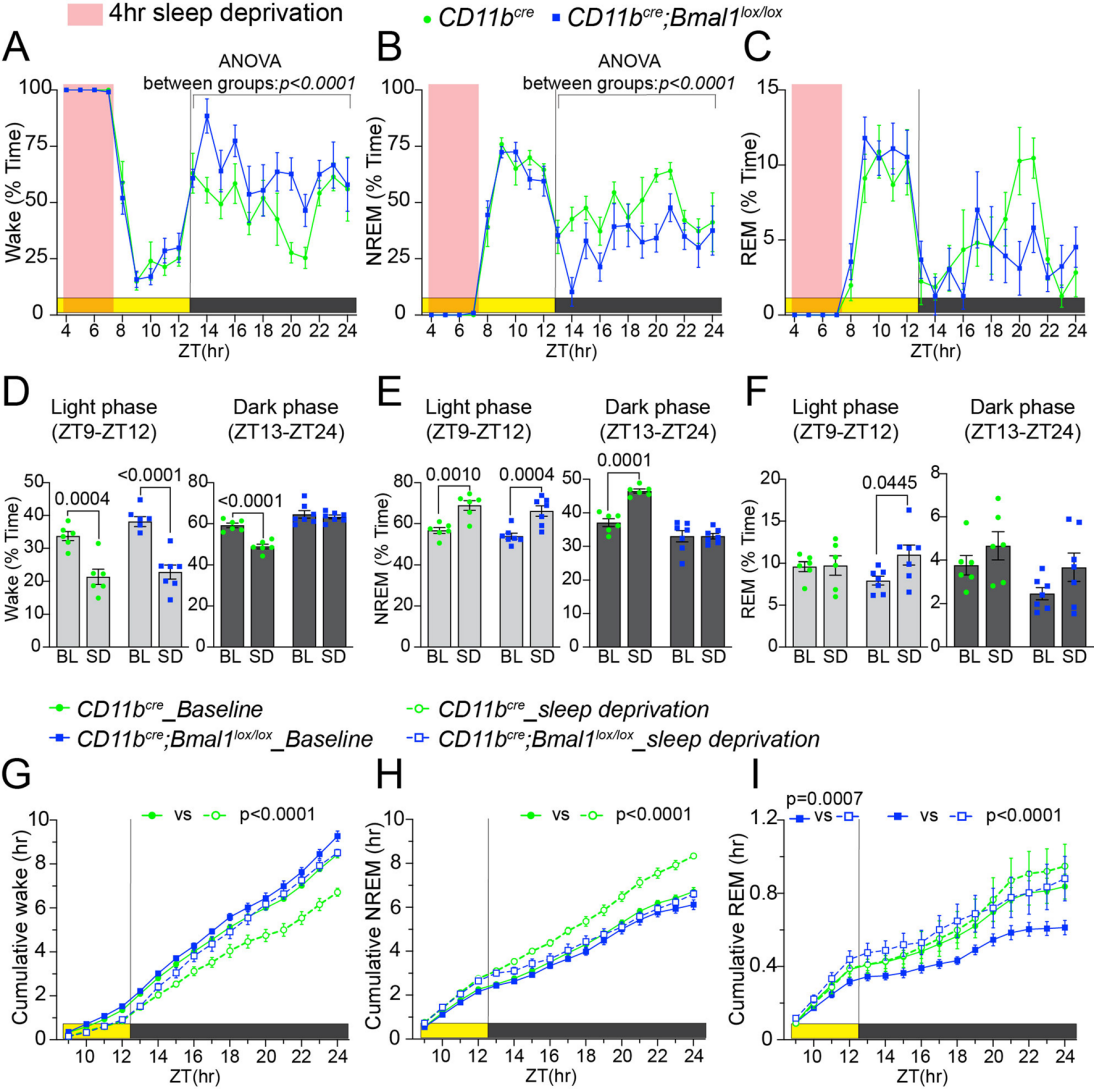
Microglial BMAL1 deficiency alters recovery from sleep deprivation. **A–C** Percent of time per hour spent in wake (**A)** NREM sleep (**B)** and REM sleep (**C)** during a 4-h sleep deprivation (pink) and subsequent recovery during the remaining light phase and full 12-h dark phase in aged *CD11b*^*cre*^ (18–20 months; *n* = 6) and *CD11b*^*cre*^*;Bmal1*^*lox/lox*^ (*n* = 7) mice. **D**–**F** Comparisons of the percentage of time spent in wake (**D)** NREM sleep (**E)** and REM sleep (**F)** across the entire 4-h light phase recovery period (left) and 12-h dark phase recovery period (right) during baseline (BL) and sleep deprivation (SD) recordings. **G**–**I** Cumulative amount (total hours) of wake (**G**), NREM (**H)** and REM (**I)** across the entire recovery period during both BL and SD recordings. Data are represented as the mean ± SEM. Significant between group differences in **A**, **B** are expressed as *P*-values calculated using two-way ANOVA. All significant within group differences between baseline (BL) and sleep deprivation (SD) are expressed as adjusted *P*-values generated from Sidak’s post hoc analysis following two-way ANOVA. *ZT* zeitgeber time

Core clock genes influence compensatory mechanisms induced by sleep deprivation [53, 60]. Given that the loss of *Bmal1* in microglia in aged mice was sufficient to disrupt the sleep–wake cycle and change sleep architecture, we explored the effects of sleep deprivation in these mice. We subjected aged *Bmal1* cKO and WT mice to a 4-h sleep deprivation protocol during the non-active light phase/subjective night and then assessed sleep recovery. During the light phase recovery period, a rebound effect was observed in both the WT and *Bmal1* cKO mice, with both groups spending less time awake (Fig. 9A and D; WT: *p* = *0.0004;* Bmal1 cKO: *p* < *0.0001*) and more time in NREM sleep (Fig. 9B and E; WT: *p* = *0.0010;* Bmal1 cKO: *p* = *0.0004*) compared to their baseline levels from the same timeframe. An increase in the time spent in REM sleep was observed in aged *Bmal1* cKO mice (Fig. 9C and F; *p* = *0.0445)* compared to WT mice. During the dark phase recovery period, WT mice continued to spend less time awake (Fig. 9A and D*; p* < *0.0001*) and more time in NREM sleep (Fig. 9B and E; *p* = *0.0001*) compared to baseline, whereas the amount of time *Bmal1* cKO mice spent in wake, NREM, and REM sleep did not differ from baseline (Fig. 9A and F).

The cumulative amount of time spent in wake, NREM, and REM sleep did not differ between groups during the light phase recovery period (Fig. 9G, H). However, during the dark phase recovery period, the cumulative time spent by WT mice in wake was decreased (Fig. 9G, H; *p* < *0.0001*), and increased in NREM (Fig. 9G, H; *p* < *0.0001*). Unlike WT mice, *Bmal1* cKO mice exhibited no compensatory changes to sleep deprivation in wake and NREM states (Fig. 9G, H). While the cumulative time spent by WT mice in REM did not differ from baseline after sleep deprivation in both the light and dark phase recovery periods, the cumulative time spent in REM was significantly increased from baseline in *Bmal1* cKO mice during the light phase (Fig. 9I; *p* = *0.0007*) and dark phase (Fig. 9I; *p* < *0.0001*) recovery periods.

Taken together, these results suggest that the homeostatic response to sleep deprivation is markedly blunted in aged *Bmal1* cKO mice.

## Discussion

The results presented here demonstrate the importance of the clock protein, BMAL1, in microglial function in aging, and highlight the importance of homeostatic circadian interactions between microglia and neurons. Here, we show that with aging, microglial BMAL1 deficiency leads to deficits in non-spatial and spatial learning and memory and increased anxiety. Young *Bmal1* cKO mice performed better than WT mice in spatial learning and memory [29] but showed no other differences suggesting that with loss of *Bmal1*, compensatory mechanisms that are activated in young mice are not sustained as the mice age. In aged *Bmal1* cKO mice, electrophysiology of the CA1 hippocampal circuit demonstrated reduction of long-term hippocampal plasticity that was accompanied by a decrease in synaptic vesicle release. These data indicate that synapses in aged *Bmal1* cKO mice may not be functional, an interpretation supported by the observed increase in immature spines by Golgi staining. Additional investigation revealed that microglial BMAL1 deficiency results in reduced deposition of C1q on synapses and reduced microglial synaptic engulfment. This functional deficit may underlie the observed deficits in hippocampal function as well as the additional deficits in the sleep–wake cycle and response to sleep deprivation in aged *Bmal1* cKO mice.

Investigation of basal synaptic transmission, short- and long-term synaptic plasticity revealed dysregulated presynaptic vesicle release that reduced the magnitude of long-term hippocampal plasticity in aged *Bmal1* cKO mice. One potential mechanism for the increased PPR observed in aged *Bmal1* cKO mice may be reduced availability of presynaptic calcium that is required for neurotransmitter release. This would lead to a decrease in the readily releasable pool of vesicles and a reduction of LTP. Overall the differences in PPR and fEPSP observed between aged *Bmal1* cKO and WT mice indicate a difference in density of functional synapses within the CA1, an interpretation supported by the higher proportion of filopodial-like spines with Golgi stain in aged *Bmal1* cKO mice, and consistent with studies correlating dendritic spine density with neuronal plasticity and memory formation [61, 62]. Phosphorylated CAMKII, which facilitates induction of LTP [31, 63] by mediating the insertion of endogenous postsynaptic AMPA receptor, did not differ between aged *Bmal1* cKO and WT mice. However, levels of the AMPA receptor subunit, GluA1, which is critical to mediating LTP [35], were significantly decreased in the CA1 hippocampal region of aged *Bmal1* cKO. The reduced density of AMPA receptors at the post-synapse may explain the impaired induction and maintenance of LTP observed in the aged *Bmal1* cKO mice.

Microglia engulf non-functioning dendritic spines and play a critical role in synapse elimination and refinement of neural circuits [16, 17]. The observed increase in levels of pre- and post-synaptic proteins, immature synapses by Golgi and the reduction in engulfment of synaptic components by BMAL1*-*deficient microglia in aged mice support a critical role for microglial BMAL1 in synaptic pruning. This observation of increased dendritic spine density in aged *Bmal1* cKO mice is in contrast to increased pruning of synapses in aging and models of neurodegeneration. We observed a reduced deposition of C1q at CA1 synapses in aged *Bmal1* cKO hippocampus, where C1q promotes microglial engulfment of synaptic material [17, 41] through interaction with the microglial C3 receptor [43, 64]. Our observations suggest insufficient tagging of synapses destined for pruning in the aged *Bmal1* cKO mice and an associated increase in spine density. We also show a reciprocal increase in C3 which may be due to its accumulation in response to decreased C1q and C3 convertase levels. Our results are consistent with another study where global deletion of *Bmal1* increased levels of C3 in the brain, and deletion of REV-ERBα, a negative regulator of BMAL1 expression, increased synaptic phagocytosis [65]. Indeed a mutant amyloid precursor protein (APP) transgenic mouse model lacking C1q also demonstrated a similar increase in C3 [66]. Additionally, deficiency of the circadian clock protein, CRY1, decreases levels of C1q [67], and BMAL1 is known to bind the promoter regions of PU.1 and MafB, two transcription factors that regulate C1q expression [68–70]. Therefore, a role for BMAL1 deficiency in disrupting synaptic pruning could be explained by the loss of circadian regulation of complement proteins.

The increase in IBA1, reduced morphological complexity, and decrease in lysosomal proteins CD68, LAMP1 and p62 in aged *Bmal1* cKO mice suggest that the microglia are activated but poorly phagocytic. Transcriptomic analysis showed enrichment for innate immune pathways and a significant enrichment of genes associated with the omegasome, a transient structure required for autophagosome formation that is dependent on p62 expression [49]. One interpretation is that lysosomal dysfunction in BMAL1-deficient microglia results in failure of proper omegasome and autophagosome formation, leading to defective synaptic engulfment, an effect further compounded by the reduced C1q deposition on synapses that need to be pruned.

Microglial molecular processes and structure exhibit a daily rhythmicity that cycles with sleep–wake states, with larger soma and increased expression of CD11b, a subunit of the complement receptor, CR3, at the beginning of the sleep cycle, and smaller soma with extensive and motile processes during the wake cycle [50, 71]. Spine elimination mediated by microglia occurs during sleep, and is associated with increased C1q and C3, phagocytosis, and decreases in synaptic-associated proteins such as PSD95 and synapsin I [71]. There are sleep-dependent increases in C3, C4, and C5 that are abrogated with sleep deprivation [72] and potentially function in synapse remodeling. Our results demonstrate a profound effect of microglial deficiency of BMAL1 on the sleep–wake cycle as well as the homeostatic need for sleep after sleep deprivation. Previous studies have demonstrated altered EEG delta power dynamics in response to sleep deprivation from loss of or mutated clock genes [73]. Global deletion of *Bmal1* in mice has been shown to increase total sleep time and sleep fragmentation [53], however in that study, *Bmal1* was ablated in all cell types including neurons. New learning is encoded during the wake state and sleep consolidates and stabilizes the memory of the learned experience [74–76]. With the loss of microglial BMAL1, this process is disrupted and may account for the cognitive deficits and sleep–wake deficits observed in aged *Bmal1* cKO mice.

In conclusion, microglial BMAL1 function is critical to maintaining hippocampal plasticity and memory, the sleep–wake cycle, and sleep homeostasis in aging. BMAL1 deficiency in microglia causes profound disruptions in microglial morphology, lysosomal function and generation of C1q, an essential mediator of synaptic engulfment and pruning necessary for the maintenance of neural circuitry.

## Supplementary Information

**Additional file 1: Figure S1.** Young *Bmal1* cKO mice do not demonstrate changes in synaptic density proteins, PSD95, or SNAP25. **Figure S2.** Young *Bmal1* cKO mice do not demonstrate changes in C1q or CD68 in CA1 hippocampus. **Figure S3.** Young *Bmal1* cKO mice do not demonstrate changes in microglial activation and morphology. **Figure S4.** Microglial BMAL1 deficiency decreases LAMP1 in the CA1 hippocampal region. **Figure S5.** BMAL1 deficiency in microglial gene expression in young mice. **Figure S6.** Baseline EEG spectral power characteristics are shifted during wake period in aged *Bmal1* cKO mice. **Video S1.** Decreased engulfment of PSD95 puncta by CD68+ microglia in aged *Bmal1* cKO mice. Appendix list of antibodies.

**Additional file 2.** Functional enrichments among genes downregulated in Bmal1 cKO.

## Data Availability

The datasets used and analyzed during the current study are available from the corresponding author on reasonable request. The data generated from the transcriptomic analysis have been deposited to the GEO repository with the identifier “GSE214514”. To review go to http://www.ncbi.nlm.nih.gov/geo/query/acc.cgi?acc=GSE214514 and enter the token qpyfmoiuzfkrpgl.
